# Perinatal Exposure to Nicotine Alters Sperm RNA Profiles in Rats

**DOI:** 10.3389/fendo.2022.893863

**Published:** 2022-05-04

**Authors:** Hetan Wang, Jie Liu, Jianjun Gao, Wei Yan, Virender K. Rehan

**Affiliations:** ^1^ Department of Medical Genetics, China Medical University, Shenyang, China; ^2^ The Lundquist Institute for Biomedical Innovation at Harbor-UCLA Medical Center, Torrance, CA, United States; ^3^ David Geffen School of Medicine at University of California, Los Angeles, Los Angeles, CA, United States

**Keywords:** asthma, nicotine, smoking, lung, epigenetic inheritance, small RNA, large noncoding RNA

## Abstract

Perinatal exposure to smoking has been associated with childhood asthma, one of the most common pediatric conditions affecting millions of children globally. Of great interest, this disease phenotype appears heritable as it can persist across multiple generations even in the absence of persistent exposure to smoking in subsequent generations. Although the molecular mechanisms underlying childhood asthma induced by perinatal exposure to smoking or nicotine remain elusive, an epigenetic mechanism has been proposed, which is supported by the data from our earlier analyses on germline DNA methylation (5mC) and histone marks (H3 and H4 acetylation). To further investigate the potential epigenetic inheritance of childhood asthma induced by perinatal nicotine exposure, we profiled both large and small RNAs in the sperm of F1 male rats. Our data revealed that perinatal exposure to nicotine leads to alterations in the profiles of sperm-borne RNAs, including mRNAs and small RNAs, and that rosiglitazone, a PPARγ agonist, can attenuate the effect of nicotine and reverse the sperm-borne RNA profiles of F1 male rats to close to placebo control levels.

## Introduction

Asthma is one of the most common childhood diseases with an increasing prevalence over the past decades (1, 2). Among a multitude of potential causes, perinatal exposure to smoking has been associated with childhood asthma and a lifelong decrease in pulmonary functions in both humans and animal models (3, 4). In general, exposure to smoke constituents *in utero* and/or during early postnatal development has been regarded as the primary cause as it is well-established that the chemicals released from smoking, especially nicotine, adversely affect the developing lung, rendering increased susceptibility to childhood asthma (5–9). Interestingly, we and others have shown that childhood asthma induced by perinatal exposure to nicotine can be transmitted across multiple generations even in the absence of the same exposure (10–14). This finding is of great interest and significance because it suggests that perinatal exposure to smoking/nicotine not only causes asthma in the immediate offspring but also results in changes in their germline, leading to the transgenerational inheritance of childhood asthma.

Given that the childhood asthma induced by perinatal exposure to nicotine arises in one generation and the distribution of this disease phenotype in subsequent generations never follows Mendel’s Law (11–14), it is highly unlikely that the asthma phenotype results from genetic mutations caused by nicotine exposure. Instead, transgenerational inheritance of the phenotype is most probably mediated by an epigenetic mechanism. Both inter-and trans-generational epigenetic inheritance of disease phenotypes induced by exposure to environmental chemicals, over-or under-nutrition (e.g., high-fat diet (HFD) or starvation), or traumatic stress has been convincingly demonstrated at least in animal models (15–17). However, the underlying molecular mechanisms remain elusive. Since mammals reproduce sexually, the epigenetic codes that induce the acquired traits must lie in the gametes, sperm, and eggs. Indeed, epimutations, including changes in sperm DNA methylome (e.g., 5mC), histone modifications, and small RNA profiles, have been associated with various acquired traits in both human and animal models (18, 19). However, the causative relationship between specific epimutations (e.g., altered DNA methylation or histone marks) and specific phenotypes has not been established. Interestingly, several studies have shown that sperm total or small RNAs from male mice with an epigenetic phenotype (e.g., metabolic disorders induced by HFD and the whitetail tips caused by *Kit* paramutation), seem capable of inducing a similar phenotype in offspring derived from zygotes injected with either total or small RNAs isolated from the sperm, suggesting that sperm RNAs may function as the epigenetic codes responsible for the paternal transmission of certain acquired traits (19–21). Our previous studies have shown that the sperm 5mC profiles and histone marks are altered in the male rats with perinatal exposure to nicotine (11, 22). Given that both DNA methylation and histone marks in sperm are largely established during testicular development and spermatogenesis (23, 24), it is plausible to hypothesize that both large and small sperm-borne RNAs may also be altered in the male rats of our perinatal nicotine rat models.

Here, we report that indeed, both mRNA and small RNA transcriptomes were altered in the sperm of F1 male offspring of F0 dams with the perinatal treatment of nicotine. Consistent with our earlier reports (25, 26), we also found that a PPARγ agonist could attenuate the effects of perinatal exposure to nicotine on sperm RNA profiles in the F1 male offspring.

## Materials and Methods

### The Perinatal Nicotine Exposure Rat Model

The perinatal nicotine exposure rat model used in this study was established as described previously (12, 22, 27, 28). Briefly, time of mating-matched, first-time pregnant, pair-fed adult (2 months of age) Sprague Dawley rat dams (F0) with bodyweight between 200-250 g received either placebo (saline, n = 3), nicotine (1 mg/kg, subcutaneously, n = 3), or nicotine (1 mg/kg, subcutaneously) plus rosiglitazone (RGZ) (3 mg/kg, intraperitoneally, n = 3) in 100 μL volumes once daily from embryonic day 6 (E6) of gestation to postnatal day 21 (PND21). The dose of nicotine used (1 mg/kg/day) is within the range of nicotine exposure in moderately heavy smokers (29–31). At this dose, the pulmonary structural, molecular, and functional changes that we observed in the rat model used are similar to those demonstrated in numerous other perinatal nicotine and smoke exposure models (12, 27, 32–35). Animals were maintained in a 12h-light and 12h-dark cycle, pair-fed according to the previous day’s food consumption by the nicotine-treated group and were allowed free access to water. Following spontaneous delivery at term, the F1 pups were allowed to breastfeed ad libitum. At PND21, pups were weaned and maintained in separate cages. At PND60, males [n = 3 (from 3 separate litters) for each group] were euthanized by pentobarbital overdose injected intraperitoneally, followed by epididymis collections as quickly as possible. The epididymides were kept in ice-cold F12 culture medium until sperm isolation within 1-2 hours of the collection, as outlined below. All animal procedures were performed following the National Institutes of Health guidelines for the care and use of laboratory animals and approved by the Institutional Animal Care and Use Committee at The Lundquist Institute for Biomedical Innovation at Harbor-UCLA Medical Center.

### Collection and Purification of Sperm Cells

At culling, each epididymis was isolated by cutting the vas deferens and muscle connections with the testis. After trimming the surrounding connective tissue, the two epididymides from each animal were placed in a tissue culture plate containing 3 mL of HTF culture medium (Sigma, EmbryoMax^®^ Human Tubal Fluid (HTF) (1X), Cat No. MR-070-D) on ice. The spermatozoa were released into the culture media by making 6-8 small cuts to each epididymis with a sharp blade, and the plates were placed in a culture incubator at 37°C for 30 minutes. Following incubation, the medium containing spermatozoa was filtered through a cell strainer (Genesee Scientific, 70 μm Advanced Cell Strainers, Cat No. 25-376) to a 50 mL conical tube, and the filtrate was divided into four 1.5 mL micro-centrifuge tubes. The tubes were centrifuged at 1000×g for 5 minutes, supernatants discarded, and 1 mL lysis buffer (0.05% SDS and 0.005% Triton X-100 in distilled water) added to each tube to gently suspend the pellet. The tubes were kept on ice for 5 minutes to lyse and remove the somatic cell contamination. After confirming the purity of isolated sperms microscopically, the samples were centrifuged at 3000×g for 5 minutes. The supernatants were discarded, and each pellet gently suspended in 1 mL ice-cold PBS. The suspensions from two tubes were pooled and centrifuged at 3000×g for 5 minutes. The supernatants were discarded, and pellets stored at -80°C until RNA isolation and establishment of cDNA library.

### Total RNA Extraction

Sperm samples were pooled (n = 3 mice/biological replicate) and subjected to RNA extraction for RNA-seq, as described below. RNA was extracted from cells according to the manufacturer’s instructions using the mirVana miRNA Isolation Kit (ThermoFisher, Cat No. AM1560). The Qubit RNA High Sensitivity Assay Kit (Invitrogen, Cat No. Q32855) was used to quantify the extracted RNA and measured on the Qubit 2.0 Fluorometer (Invitrogen).

### Large RNA Libraries Construction

Large RNA libraries were constructed using KAPA Stranded RNA-seq Kit with RiboErase (Roche, Cat No. KK8484) according to the manufacturer’s instructions, as described previously (36), and sequenced using HiSeq 2500 system for paired-end 50 bp sequencing.

### Large RNA-Seq Data Analysis

The following workflow was used in bioinformatic analyses of the RNA-seq data: QC check (fastQC) ➔ alignment (Hisat2) ➔ featureCounts (subread) ➔ Differential gene expression analysis (DESeq2) ➔ Pathway Enrichment, GO analysis (Bioconductor clusterProfiler). To ensure the quality of RNA-seq data, fastq files were subjected to fastQC (37) to check their quality and changes after adaptor and quality trim. MultiQC (38) was then utilized to analyze and integrate the QC reports (**Figure S1**). HISAT2 was used to perform alignment (39). Each sample yielded a bam file after being aligned to the genome. Feature counts from each bam file that map to the genomic features in the provided annotation file was realized by subread function (40). DESeq2 was used to analyze the gene differential expression (41). Markers/genes with the sum of reading count across all cases and controls at 10 or greater were kept for further analyses. To interpret the expression data, a universal enrichment tool named “clusterProfiler” was applied to infer gene set enrichment (42).

### Annotation of lncRNAs From Large RNA-Seq Data

LncRNA information was first obtained with gene symbols by merging two Ensembl releases (release-105 Rattus_norvegicus.mRatBN7.2.ncRNA.fa.gz and release-104 Rattus_norvegicus.Rnor_6.0.104.gtf.gz). The gene symbols were then used in differential expression analyses as keywords to search in Ensemble to obtain the lncRNA information that can be annotated. Finally, those two are combined to obtain all the lncRNA gene symbols. The extracted padj of these lncRNAs were much larger than 0.05.

### Small RNA Libraries Construction

Small RNA libraries were constructed using NEBNext^®^ Small RNA Library Prep Set for Illumina^®^ (Multiplex Compatible) (NEB, Cat No. E7330L) according to the manufacturer’s instructions, as described previously (43), and sequenced using HiSeq 2500 system for single-end 50 bp sequencing.

### Small RNA-Seq Data Analysis

Cutadapt (44) was used to remove adaptors and trim low quality reads (q > 20). The fastq files after QC filter were used to run the AASRA pipeline using default parameters (45). Eight small species, incuding miRNA, tRNA, piRNA, rRNA, snRNA, snoRNA, Mt_rRNA and Mt_tRNA, were annotated. The subsequent analyses using Featurecounts and DESeq2 were performed the same as large RNA-Seq. TargetScan was used to identify potential miRNA targets, the candidate target genes were used for gene ontology (GO) enrichment analyses using “clusterProfiler”.

### qPCR Analysis

cDNAs for large and small RNA were prepared as previously described (43). Briefly, large RNAs were reverse transcribed to cDNAs using SuperScriptTM II Reverse Transcriptase (Thermo Fisher Scientific, Cat No. 18064014). Then qPCR analyses for large RNA were conducted using Fast SYBR^®^ Green Master Mix (Thermo Fisher Scientific, Cat No. 4385616). Gapdh was used for large RNA expression normalization. Small RNAs were poly(A) tailed using E. coli Poly(A) Polymerase (NEB, Cat No. M0276L) followed by reverse transcription with LD_CDS primer using SuperScript™ II Reverse Transcriptase. qPCR analyses for small RNA were then performed using TaqMan™ Gene Expression Master Mix (Thermo Fisher Scientific, Cat No. 4369016) with Illu lib quant probe. U6 was used for small RNA expression normalization. The primer sequences used in this study for qPCR are listed in Datasets S1.

### Statistical Analysis

All data were subjected to statistical analysis using the SPSS program (IBM, SPSS, New York, NY, USA) and shown as mean ± standard error of the mean (SEM). And statistical differences between two groups were assessed by two samples *t*-test. Symbols *, ** and *** represent p < 0.05, p < 0.01 and p < 0.001, respectively.

### Availability of Data and Materials

The RNA-seq data have been deposited into the National Center for Biotechnology Information Sequence Read Achieve database (accession no. PRJNA813596).

## Results

### mRNA Profiles in Sperm From the Male Rats Born to Control, Nicotine-Treated, and Nicotine Plus RGZ-Treated Dams

**Figure 1:**
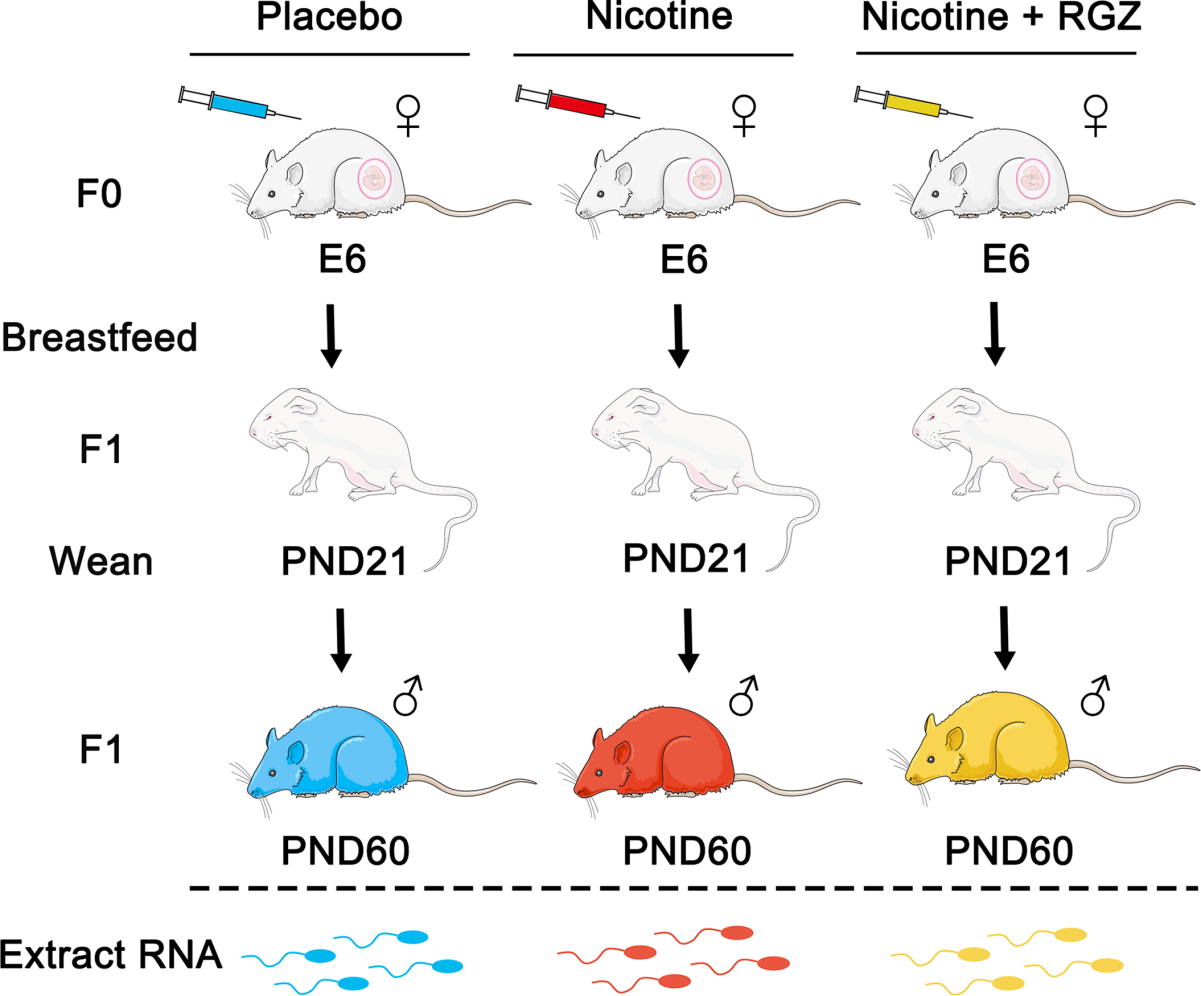
The perinatal nicotine exposure rat model used in the present study. Dams (F0) received either placebo (saline), nicotine (1 mg/kg, subcutaneously), or nicotine (1 mg/kg, subcutaneously) plus rosiglitazone (RGZ) (3 mg/kg, intraperitoneally) once daily from embryonic day 6 (E6) of gestation to postnatal day 21 (PND21). The F1 pups were allowed to breastfeed ad libitum. At PND21, the F1s were weaned and maintained in separate cages. Pure sperm cells of F1 male rats were collected at PND60 and sperm RNA was extracted and used for RNA-seq analyses.

**Figure 2:**
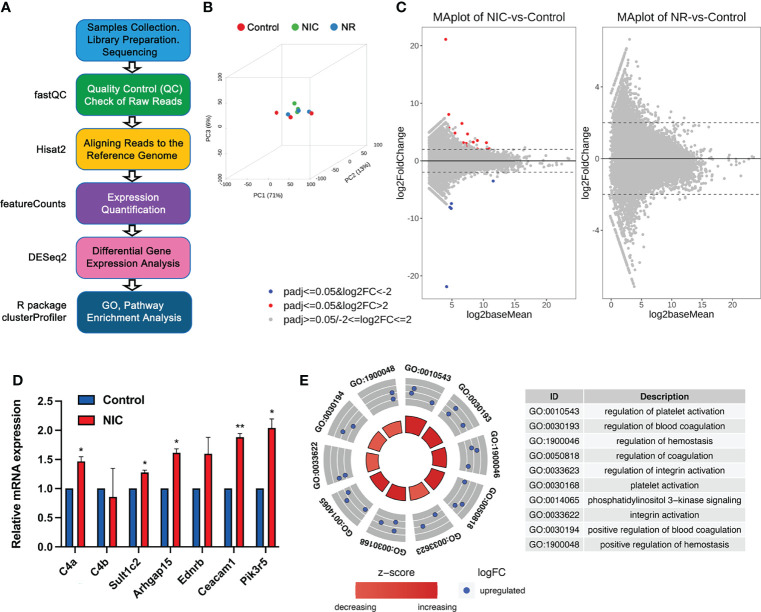
RNA-seq analyses of sperm mRNA profiles in placebo, nicotine-treated and nicotine plus RGZ-treated F1 male rats. **(A)** The workflow for large RNA-seq data analyses, showing the major steps and bioinformatic tools used in the study. **(B)** Three-dimensional principal component analyses of the large RNA-seq data from nicotine-treated, nicotine plus RGZ-treated, and placebo-treated sperm samples. **(C)** MA plots showing differentially expressed genes (upDEGs and downDEGs) detected between nicotine-treated and placebo-treated sperm samples (left panel) and between nicotine plus RGZ-treated and placebo-treated sperm samples (right panel). The Log2baseMean represent the Log2 mean value of DESeq2 normalized counts between nicotine-treated and placebo-treated sperm, or between nicotine plus RGZ-treated and placebo-treated sperm. Log2 fold change (Log2FC) was calculated by the Log2 mRNA counts of nicotine-treated sperm/placebo-treated sperm, or nicotine plus RGZ-treated sperm/placebo-treated sperm. Genes that pass a threshold of padj <= 0.05, log2FC > 2 and padj <= 0.05, log2FC < -2 in differential expression analysis were designated by red (up-regulated) and blue (down-regulated) in nicotine-treated or nicotine plus RGZ-treated sperm relative to placebo-treated control sperm cells. **(D)** qPCR validation of mRNA expression levels in placebo and nicotine-treated sperm. *Gapdh* was used as an internal control. Data are presented as mean ± SEM (n = 3). **P < 0.01; *P < 0.05. **(E)** Circle plots showing the top 10 Gene Ontology (GO) terms of biological process analyzed from 16 significantly dysregulated genes in sperm samples from rats injected with nicotine compared to those from rats injected with placebo.

Adult female rats (F0 dams) received placebo (saline subcutaneously as controls), nicotine (1 mg/kg, B.W. subcutaneously), or nicotine (1 mg/kg, B.W. subcutaneously) plus RGZ (3 mg/kg, B.W., intraperitoneally) between E6 and PND21 (**Figure 1**). Cauda epididymal spermatozoa of F1 male offspring (n = 3, from 3 separate litters in each group) were collected at PND60 and used for large RNA deep sequencing (RNA-seq), followed by bioinformatics analyses using the pipeline as illustrated (**Figure 2A**).

The 3D principal component analyses (3D-PCAs) verified that the differential transcriptomes of placebo control, nicotine-treated (NIC) and nicotine plus RGZ-treated (NR) sperm samples were indeed clustered separately (**Figure 2B**). A total of 29 differentially expressed mRNAs (21 upregulated and 8 downregulated mRNAs) satisfied the criteria of padj (adjusted p-value) less than 0.05, and fold change greater than 0.2 and less than −0.2 (logFC ± 0.2) in NIC sperm samples compared to placebo controls (Datasets S2). The MA plots (**Figure 2C**, left panel) illustrate the differentially expressed genes (DEGs). In contrast, no significantly dysregulated mRNAs (padj <= 0.05 and |log2FC| ≥ 2) were detected in sperm of the control and NR groups (**Figure 2C**, right panel and Datasets S3).

Many of the upregulated genes in nicotine-treated sperm are known to be involved in asthma pathogenesis, including *L-Histidine decarboxylase (Hdc)*, *Fc receptor-like 3 (Fcrl3)*, *Endothelin receptor type B (Ednrb)*, and *Complement C4A (C4a)*. *Hdc* encodes a unique enzyme in mammals which catalyzes histamine formation from L-histidine and histamine plays a critical role in the pathogenesis of bronchial asthma. In particular, the level of *Hdc* mRNA is elevated in asthmatic patients (46). Furthermore, *Hdc* allele Glu644 in homozygotes increases the risk of rhinitis in the study population, supporting a prominent role for genetic variants associated with histamine homeostasis in developing allergic disease risk (47). In studying a single nucleotide polymorphism (SNP) in *Fcrl3* in asthma and/or AR patients and healthy controls in a Chinese Han population, novel SNP rs7528684 appears to be associated with asthma with comorbid AR, and *Fcrl3_3* (rs7528684) and *Fcrl3_6* (rs3761959) SNPs are protective against asthma in Mexican male patients (48, 49). As the receptor for asthma related gene *EDN1*, the 30G>A SNP in *Ednrb* is strongly associated with the degree of airway obstruction, especially in patients with factors that induce airway remodeling, such as asthma or smoking (50). And in the murine model of asthma, *Ednrb* receptor antagonists is found to effectively inhibit allergic reactions (51). When compared with the children without asthma, an increasing serum level of *C4* component of the complement system is observed in the majority of the patients with intermittent atopic asthma, representing a biomarker for diagnosis of intermittent atopic asthma (52). In addition, the level of *C4a* increases in the plasma of patients with aspirin-induced asthma, and significantly correlated with FEV1 (53).

However, several genes are newly implicated in asthma, including *Rho GTPase activating protein 15 (Arhgap15)*, *Pleckstrin (Plek)*, and *Transcription factor EC (Tfec)*. *Arhgap15* has been called a ‘‘master negative regulator of neutrophil functions’’, and validated as a differentially expressed novel transcript in patients with asthma (54, 55). *PLEK* is a major substrate for protein kinase C signaling, a pathway strongly implicated in asthma pathogenesis was upregulated in severe asthmatics and exhibited a moderate ability to distinguish between severe and mild-moderate asthmatics (56). Furthermore, earlier studies have revealed an *IL-4/STAT-6/Tfec/IL-4Rα* positive feedback regulatory loop, in which *Tfec* transcribes *IL-4Rα* expression to promote M2 programming in macrophages, which was implicated in asthma pathogenesis (57). In addition, several genes were involved in lung cancer and other lung diseases, such as *Ceacam1* (58, 59), *Ereg* (60, 61)*, Selp* (62, 63), and *Pik3r5* (64, 65). The most conspicuous genes among downregulated ones are members of the keratins (KRTs) and keratin-associated proteins (KRTAPs), including *Krt34*, *Krtap3-1*, and *Krtap7-1*, which are important for epidermal development and hair follicle morphogenesis (66, 67), respectively. qPCR analyses of seven dysregulated genes, including *C4a*, *C4b*, *Sult1c2*, *Arhgap15*, *Ednrb*, *Ceacam1*, and *Pik3r5*, further validated the RNA-seq data (**Figure 2D**). In addition, bioinformatic analyses showed no long non-coding RNAs (lncRNAs) were significantly dysregulated (padj <= 0.05) in sperm samples from the three groups (**Figure S2**, Datasets S4 and S5). To further understand the functions of these DEGs, gene ontology (GO) term enrichment analyses were performed, and the dysregulated genes appeared to be involved in integrin activation and platelet activation (**Figure 2E**, Datasets S6).

Taken together, perinatal exposure of nicotine appears to induce altered profiles of sperm mRNAs, but not those of lncRNAs; however, administration of RGZ appeared to attenuate the nicotine effects on the sperm mRNA profiles.

### Profiles of Small Non-Coding RNAs (sncRNAs) in Sperm From the Male Rats Born to Control, Nicotine-Treated, and Nicotine Plus RGZ-Treated Dams

**Figure 3:**
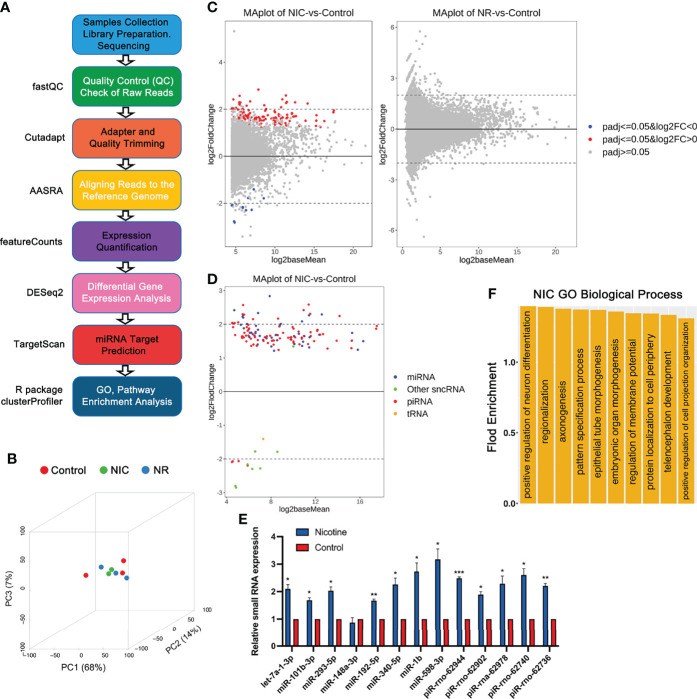
RNA-seq analyses of small non-coding RNAs (sncRNAs) in placebo, nicotine-treated and nicotine plus RGZ-treated sperm samples. **(A)** The workflow for small RNA-seq data analysis, including the major steps and bioinformatics tools used in the study. **(B)** Three-dimensional principal component analyses of the small RNA-seq data from nicotine-treated, nicotine plus RGZ-treated, and placebo-treated sperm samples. **(C)** MA plots showing the differentially expressed sncRNAs detected between nicotine-treated and placebo-treated sperm samples (left panel) and between nicotine plus RGZ-treated and placebo-treated sperm samples (right panel). The Log2baseMean represent the Log2 mean value of DESeq2 normalized counts between nicotine-treated and placebo-treated sperm. Log2 fold change (Log2FC) was calculated by the Log2 sncRNA counts of nicotine-treated sperm/placebo-treated sperm. SncRNAs that pass a threshold of padj <= 0.05, log2FC > 0 and padj <= 0.05, log2FC < 0 in differential expression analysis were designated by red (up-regulated) and blue (down-regulated) in nicotine-treated or nicotine plus RGZ-treated sperm relative to placebo-treated control sperm cells. **(D)** MA plots showing the number of significantly differentially expressed sncRNAs (padj <= 0.05) between nicotine-treated and placebo-treated sperm samples. **(E)** qPCR validation of sncRNA expression levels in placebo and nicotine-treated sperm. U6 was used as an internal control. Data are presented as mean ± SEM (n = 3). ***P < 0.001; **P < 0.01; *P < 0.05. **(F)** GO term enrichment analyses of potential target genes of significantly dysregulated miRNAs in nicotine-treated sperm. Outputs (biological processes) are sorted and plotted against fold enrichment.

To determine the effects of perinatal exposure to nicotine and nicotine plus RGZ on the sperm sncRNAs profiles of F1 males, sperm small RNAs were isolated and sncRNAs-seq was performed followed by bioinformatics analyses using AASRA (68) (**Figure 3A**). A total of eight sncRNA species, including miRNAs, mitochondrial DNA-encoded rRNA (Mt_rRNA) and tRNA (Mt_tRNA) fragments, piRNAs, rRNA fragments, tRNA fragments, snoRNAs, and snRNAs, were annotated and their normalized total counts were compared among the three groups (**Figure S3**).

Principal component analyses verified that the differential transcriptomes of the three groups were clustered separately (**Figure 3B**). A total of 139 sncRNAs were identified to be significantly dysregulated (padj <= 0.05 and |log2FC| ≥ 0 between sperm from NIC and control groups (**Figure 3C**, left panel and Datasets S7). These dysregulated sncRNAs included 47 miRNAs, 83 piRNAs, 1 tRNA, and 8 other sncRNAs. In contrast, no significantly dysregulated sncRNAs (padj <= 0.05) were detected between sperm from NR and placebo control groups (**Figure 3C**, right panel and Datasets S8). All of the dysregulated miRNAs and the vast majority (79 out of 83) of the dysregulated piRNAs were upregulated between NIC and control sperm (**Figure 3D**). Interestingly, while miRNA and piRNA levels were upregulated, other sncRNAs were mostly downregulated in nicotine-treated sperm. To validate the sncRNAs-seq data, we performed qPCR analyses on eight miRNAs (let-7a-1-3p, miR-101b-3p, 293-5p, 148-3p, 192-5p, 340-5p, 1b, and 598-3p) and five piRNAs (piR-rno-62944, rno-62902, rna-62978, rno-62740, rno-62736) in nicotine-treated and placebo control sperm. The results showed that levels of miRNAs and piRNAs were much higher in nicotine-treated sperm compared to controls (**Figure 3E**). Together, perinatal exposure to nicotine appears to alter the sncRNAs profiles, and this effect can be abolished by RGZ.

Given that miRNAs are known to function as a post-transcriptional regulator by targeting the 3’UTRs of mRNAs, we further determined the potential targets of the 47 significantly dysregulated miRNAs (Datasets S9 and S10) using TargetScan (69). Those target genes included those previously implicated in asthma pathogenesis, such as *ADAM metallopeptidase domain 33 (Adam33)*, *PHD finger protein 11 (Phf11)*, *Dipeptidyl peptidase like 10 (Dpp10)*, *Interleukin 4 (Il4)*, *Brain-derived neurotrophic factor (Bdnf)*, *Serine peptidase inhibitor, Kazal type 5 (Spink5)*, *Cd69 molecule (Cd69)*, etc. Following linkage studies, *Adam33* (70), *Phf11* (71) and *Dpp10* (72) have been identified to be associated with asthma and asthma-related phenotypes. Studies showed that *IL4*, a key effector Th2 cytokine in allergic asthma, was essential for B cells autophagy induction *in vivo* and *in vitro*, thereby further sustaining B cell survival and enhanced B cell antigen presentation (73). *BDNF* may contribute to normal lung function and immune response and may serve as a potential peripheral biomarker for asthma, especially that is aspirin-sensitive (74). Studies have shown that *SPINK5* has biological actions other than protease inhibition, which may be related to the pathogenesis of asthma (75). *CD69* was known as an early activation marker antigen of lymphocytes, had a crucial role in the pathogenesis of arthritis and allergic airway inflammation and could be a possible therapeutic target for arthritis and asthma in human patients (76). Furthermore, many of the target genes were known to be involved in other lung diseases, including *High mobility group AT-hook 2 (Hmga2)* (77), *Ubiquitin-conjugating enzyme E2C (Ube2c)* (78), *Adrenoceptor beta 3 (Adrb3)* (79), *Coronin 1C (Coro1c)* (80), *Sp1 transcription factor (Sp1)* (81), *Ras homolog family member B (Rhob)* (82), *Serum/glucocorticoid regulated kinase 1 (Sgk1)* (83), *BTG anti-proliferation factor 2 (Btg2)* (84), *Homeobox D8 (Hoxd8)* (85), *Bone morphogenetic protein 4 (Bmp4)* (86), *Protein regulator of cytokinesis 1(Prc1)* (87), etc.

GO term enrichment analyses identified that the affected target genes were mostly involved in the biological processes including embryonic organ morphogenesis, regionalization, epithelial tube morphogenesis, positive regulation of neuron differentiation, telencephalon development, protein localization to the cell periphery, pattern specification process, axonogenesis, regulation of membrane potential, and positive regulation of cell projection organization (**Figure 3F**, Datasets S11).

## Discussion

Epidemiological studies have shown that grandma’s smoking when pregnant with the mother increases the risk of asthma in the grandchild independent of the mother’s smoking status, suggesting a potential transgenerational effect of perinatal smoking on the incidence of childhood asthma (88, 89). However, considering many confounding factors, this notion remains highly correlative. Given that it would take decades to follow up on multiple generations on any transgenerational effect, we and others have developed animal models to demonstrate that childhood asthma induced by perinatal exposure to nicotine in F0 dams can persist for at least three generations in the absence of continuous perinatal exposure to nicotine in F1-F3 (11, 12). Such intergenerational and transgenerational transmission of the induced disease phenotype must be mediated by the gametes (sperm and eggs) given sexual reproduction. Indeed, our earlier data have shown that both histone marks and DNA methylation (5mC) patterns are altered in F1 sperm (10). Since the sperm DNA methylation patterns are largely established during fetal testicular development and further modified during spermatogenesis, the DMRs in F1 male rat sperm must have arisen in pro-spermatogonia and/or the subsequent spermatogenic cells including spermatogonia, spermatocytes, or spermatids. Since DNA methylation changes affect gene expression, it is possible that the mRNAs that are produced in spermatogenic cells and packed into the sperm nuclei might be altered as well. In contrast, the vast majority of nuclear histones are replaced by transition proteins and ultimately by protamine during the elongation of round spermatids (20, 90). Consequently, only trace amounts of histones (<1% in rodents and <5% in humans) are retained in sperm (91). Therefore, altered histone levels and chemical modifications must have occurred during late spermiogenesis. Since both large and small RNAs are believed to be packed into the condensing nuclei of spermatids upon elongation during spermiogenesis, the altered histone profiles may also indicate altered mRNA and small RNAs that are packed into the nuclei of sperm. Indeed, our data clearly show that the mRNA and small RNA profiles are indeed altered in the sperm of F1 male rats born to dams with perinatal exposure to nicotine.

Sperm-borne mRNAs are delivered to the oocytes during fertilization (92). It remains unclear whether these mRNAs are functional and thus necessary for fertilization and early embryonic development. Small RNAs have been detected in sperm nuclei, and miRNAs and endo-siRNAs have been shown to be essential for fertilization and early embryonic development, most likely through functional as post-transcriptional regulators (93, 94). Increasing lines of evidence also suggest that both sperm-borne large and small RNAs may have a role in mediating epigenetic inheritance of acquired traits (19). This notion is largely based on the observations that injection of either total RNA or small RNAs isolated from male mice with the specific acquired traits (e.g., metabolic disorders induced by HFD, stress response conditioned to specific odor, wound healing response conditioned to repeated liver injury, etc.) into wildtype oocytes can produce offspring displaying a similar phenotype. However, the exact molecular actions of these sperm-borne RNAs remain elusive. In the present study, we identified 29 differentially expressed mRNAs in nicotine exposed F1 male rats compared to placebo control male rats. These DEGs may represent the consequences of the dysregulated epigenome, as reflected by numerous DMRs and aberrant histone marks detected (11, 22), in spermatogenic cells during spermatogenesis. An alternative function would be that these sperm-borne mRNAs, once delivered into the cytoplasm of the oocytes, can produce proteins that participate in early embryonic development. Given that these F1 male rats all have normal fertility, the changed levels of the proteins encoded by these DEGs must be compatible with successful fertilization and embryonic development. However, it remains unknown whether these proteins can be involved in epigenetic regulations that can lead to childhood asthma. Among the differentially expressed small RNAs, miRNAs and piRNAs appear to be the dominant small RNA species in the nicotine exposed F1 male rats. miRNAs are known to function as a post-transcriptional regulator by binding the 3’UTR of mRNAs to control mRNA stability and translational efficiency (95). Sperm-borne piRNAs are largely produced in spermatocytes and spermatids, and these piRNAs are believed to control the timely degradation of mRNAs during late spermiogenesis (96, 97). It remains unclear how miRNAs and piRNAs function as carriers of epigenetic information in sperm although both have been shown to be involved in the transmission of acquired traits inter-or trans-generationally. Several studies have shown that microinjection of sperm total or small RNAs (total, miRNAs, tsRNAs) isolated from the male mice with acquired traits can induce similar phenotypes in offspring although the phenotypic penetrance varies (98–102). It would be of great interest to see whether injection of the dysregulated small RNAs in male F1 rats with perinatal exposure to nicotine also transmits the asthma phenotype to the subsequent progeny. Moreover, examination of the epigenome of the F1 lung tissue in both nicotine-exposed and placebo control males during fetal and postnatal development may shed light on the effects of the dysregulated sperm small RNAs in the future.

Rosiglitazone is a PPARγ agonist that has shown a beneficial effect in both mice and humans with asthma (103, 104). In asthmatic mice and patients, PPARγ activation appears to inhibit airway inflammation and remodeling by downregulating proinflammatory gene expression and inflammatory cell functions (105). In our rat model of childhood asthma, induced by perinatal nicotine exposure, RGZ administered in conjunction with nicotine attenuates the development of asthma (25, 26). More interestingly, the altered levels of 5mC and several histone modifications including H3 acetylation and H4 acetylation also get reversed in the lung and gonad of F1 rats (11, 22). These data suggest that RGZ has an epigenetic effect on both the target tissue (i.e., lung) and germ cells, which can largely restore the gene networks required for normal airway functions. Consistent with these previous data, our RNA-seq analyses of total RNA expression profiles in the sperm of F1 male rats also show that RGZ can attenuate the adverse effects of perinatal exposure to nicotine on the sperm RNA profiles. The effect may directly affect the expression of certain mRNAs and small RNAs. Alternatively, the altered transcriptomes may result from RGZ’s effect on DNA methylation and/or histone modifications. Nevertheless, the fact that a PPARγ agonist attenuates the effect of nicotine on sperm large and small RNA transcriptome further supports the notion that PPARγ agonists is a promising class of drugs for treating childhood asthma.

In summary, we report here that perinatal exposure to nicotine leads to alterations in the profiles of sperm-borne RNAs, including mRNAs and small RNAs, and that rosiglitazone can attenuate the effect of nicotine and reverse the sperm-borne RNA profiles of F1 male rats to close to placebo control levels.

## Data Availability Statement

The datasets presented in this study can be found in online repositories. The names of the repository/repositories and accession number(s) can be found in the article/**Supplementary Material**.

## Ethics Statement

The animal study was reviewed and approved by the Institutional Animal Care and Use Committee at the Lundquist Institute for Biomedical Innovation at Harbor-UCLA Medical Center.

## Author Contributions

VR designed the research. HW and JL performed the experiments. JG performed bioinformatic analyses. HW and WY analyzed the data and wrote the manuscript. All authors contributed to the article and approved the submitted version.

## Funding

This work was supported by grants from the NIH (HD098593, HD0085506, HD099924 to WY; and HL151769, H127137, HD071731, and HL152915 to VR); the Templeton Foundation (PID: 61174 to WY); and the TRDRP (23RT-0018, 27IP-0050, and T29IR0737 to VR).

## Conflict of Interest

The authors declare that the research was conducted in the absence of any commercial or financial relationships that could be construed as a potential conflict of interest.

## Publisher’s Note

All claims expressed in this article are solely those of the authors and do not necessarily represent those of their affiliated organizations, or those of the publisher, the editors and the reviewers. Any product that may be evaluated in this article, or claim that may be made by its manufacturer, is not guaranteed or endorsed by the publisher.
