# Supplementary material for: Perinatal Exposure to Nicotine Alters Sperm RNA Profiles in Rats
Source: Front Endocrinol (Lausanne). 2022 May 4;13:893863. doi: 10.3389/fendo.2022.893863 (PMC9114732; doi:10.3389/fendo.2022.893863)
Supplement: Supplementary file 1 [file DataSheet_1.docx]

**Supplementary Figures**

**
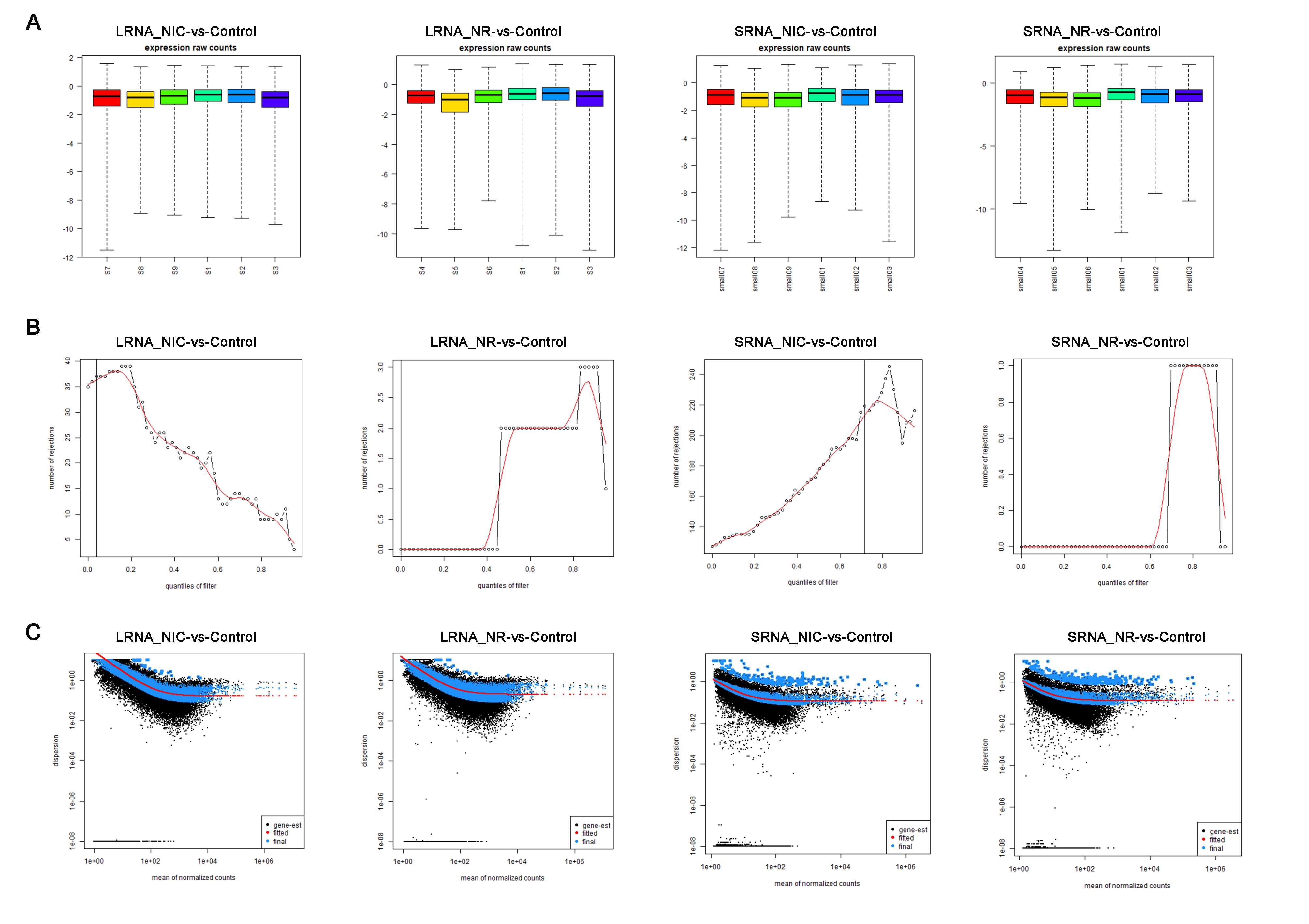
**

Fig. S1. Quality control data of large and small RNA-Seq analyses. (A) Boxplots of the Cook’s distances demonstrating the outlier for each sample as a quality control measure. (B) Threshold of independent filtering by default using the mean of normalized counts as a filter statistic, which optimizes the number of adjusted p values lower than a significance level alpha. (C) Dispersion Plots showing the estimated dispersion of the data, determining the shape of the mean-variance relationship.

Fig. S2. Heatmap showing lncRNA profiles in sperm samples from placebo control, nicotine-treated (NIC), and nicotine plus RGZ (NR)-treated male F1 rats. No significantly dysregulated lncRNAs were detected.

**
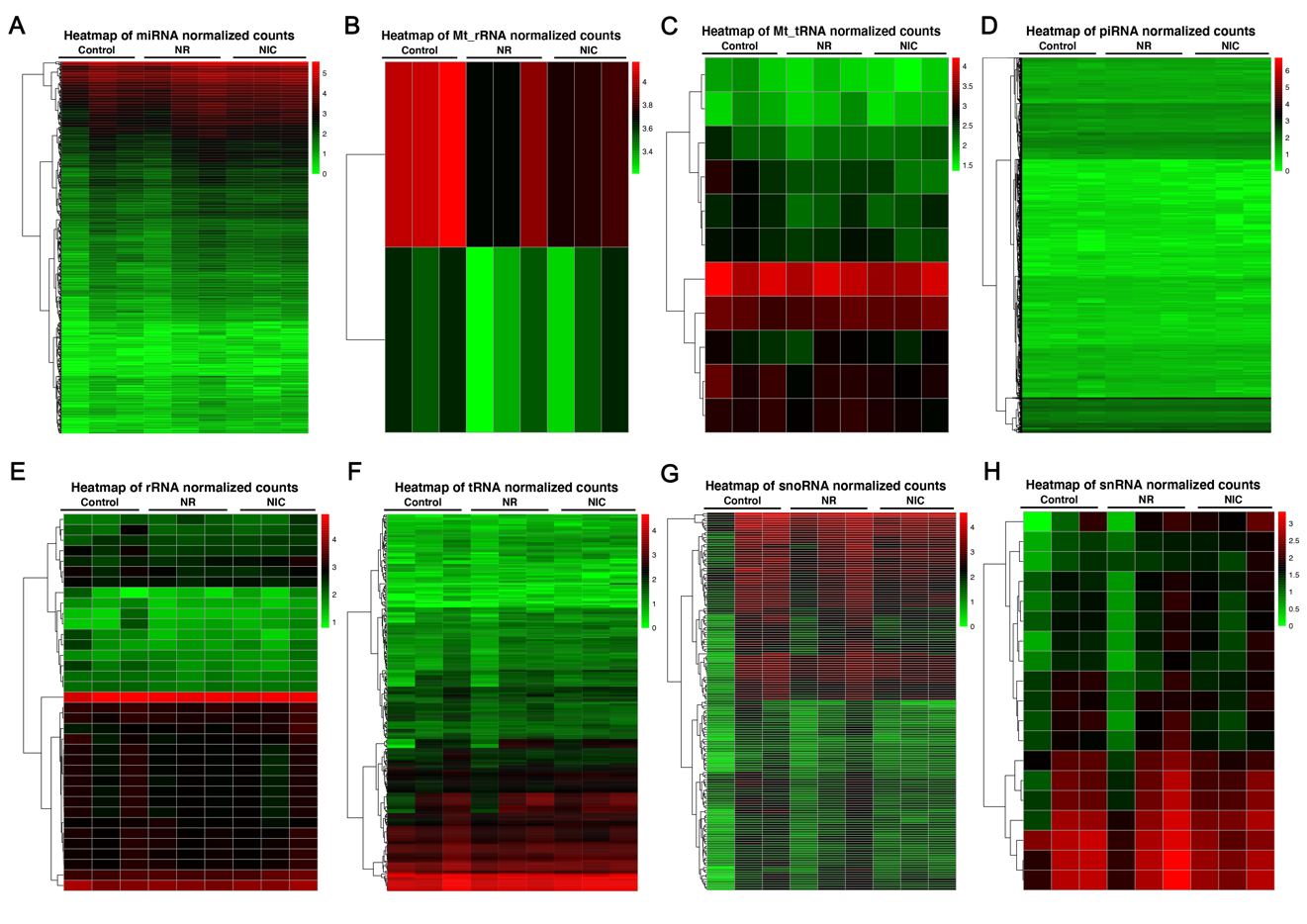
**

Fig. S3. Heatmaps showing dysregulated miRNAs (A), Mt_rRNAs (B), Mt_tRNAs (C), piRNAs (D), rRNAs (E), tRNAs (F), snoRNAs (G), and snRNAs (H) in placebo control, nicotine-treated and nicotine plus RGZ-treated sperm samples. For detailed lists of these sncRNAs, please see Datasets S8 and S9.
